# Impact of Coffee Cherry Fermentation Methods on the Quality Attributes of Dry‐Processed Coffee

**DOI:** 10.1155/tswj/4278424

**Published:** 2025-12-29

**Authors:** Mario Fernando Moncayo-Palacios, Víctor Hugo Muñoz-Carvajal, Esteban Largo-Avila, Carlos Hernán Suárez-Rodríguez, Alba Mery Garzón-García

**Affiliations:** ^1^ Servicio Nacional de Aprendizaje, Centro de Tecnologías Agroindustriales, Cartago, Valle del Cauca, Colombia; ^2^ Universidad del Valle, Caicedonia, Valle del Cauca, Colombia, univalle.edu.co

**Keywords:** carbon dioxide, chemical analysis, fermentation, natural coffee, SCA protocol, sensory analysis

## Abstract

The global demand for premium‐quality coffee is growing, as more consumers appreciate its distinct sensory attributes. Fermentation plays a key role in producing natural (dry‐processed) coffees with distinct and appealing flavors and aromas. However, improper fermentation conditions can lead to defects that negatively impact the quality of the coffee. This research aimed to compare various fermentation methods for producing natural coffees and assess their impact on both sensory attributes and chemical composition. Five fermentation processes were evaluated: exposure to air for 24 and 48 h, fermentation in sealed containers with CO_2_ for 24 and 60 h, and a control with no fermentation. The total sugar content was measured using ultra‐high‐pressure liquid chromatography (UHPLC). Fatty acids composition was analyzed using gas chromatography (GC), whereas chlorogenic acid content was determined using high‐performance liquid chromatography (HPLC). Sensory evaluation followed the Specialty Coffee Association (SCA) protocol. Results indicated that fermentation under modified atmosphere inhibited sucrose consumption, preserved chlorogenic acid levels, and reduced elaidic acid by less than 10%. Significant differences in sensory quality attributes were observed across treatments. Overall, the study concluded that fermenting coffee cherries under modified atmospheres is associated with the high sensory quality of natural coffee.

## 1. Introduction

In the pursuit of high‐quality coffees, producers often conduct various experiments, many of which focus on fermenting coffee cherries intended for natural coffee production. These tests can sometimes result in coffees with exceptional sensory qualities, leading to higher market prices. However, the lack of standardized guidelines or parameters for coffee cherries fermentation increases the risk of compromising bean quality. Fermentation is a complex biochemical process influenced by numerous variables and is regarded as the stage where most defects can occur in coffee processed via wet methods [1]. The growing demand for high‐quality, differentiated coffees has significantly influenced market dynamics and production systems, driving them to adapt to evolving consumer preferences [2]. At a commercial level, three types of coffee processing are basically recognized: dry processing, wet processing, and semidry processing [3].

Dry fermentation plays a crucial role in defining the physical, chemical, and sensory attributes of coffee. This process, deeply rooted in coffee‐producing regions, has gained growing significance due to its substantial impact on the final quality of the product. Numerous studies have explored variations in fermentation conditions, methodologies, and their effects on parameters such as pH, acidity, volatile compounds, and antioxidant activity. Cavanagh et al. [4] highlighted the operational advantages of dry fermentation, emphasizing its efficiency in reducing both time and costs. In recent years, Colombia has seen a rising interest in achieving differentiated sensory profiles, driving greater attention toward dry‐processed coffees that produce natural profiles [5]. However, many coffee fermentation processes lack adequate control, leading to the development of defects, inconsistencies in quality, and economic losses for coffee growers. Additionally, these uncontrolled processes impose limitations on accessing high‐quality coffee markets [6].

Previously, the study by Galarza and Figueroa [7] provided additional insights by evaluating the effects of different dry fermentation conditions (namely, aerobic, anaerobic, and modified atmosphere) on the development of key volatile compounds in coffee. Additionally, CO_2_ injection promotes greater uniformity in the coffee fermentation process, as sugar degradation occurs more slowly, favoring alcoholic and lactic fermentations. In contrast, when fermentation takes place in oxygen‐rich environments, sugars break down rapidly, leading to the formation of undesirable byproducts such as butyric and propionic acids. This condition also enables the participation of various microorganisms in mucilage transformation, resulting in variations in the coffee′s sensory profile due to the different secondary metabolites generated under each fermentation system [8–10].

Despite advances in coffee fermentation research, most studies have been conducted under laboratory‐controlled or wet fermentation conditions, limiting their applicability to natural (dry) processing systems [11]. There is still scarce empirical evidence on how modified‐atmosphere fermentation, particularly with CO_2_, affects the chemical and sensory quality of dry‐processed coffees under real production settings. Addressing this gap, the present study evaluates the effects of different CO_2_‐based fermentation methods on sugars, chlorogenic acids, fatty acid composition, and sensory attributes, contributing to a more comprehensive understanding of controlled fermentation in natural coffee production.

This study contributes to the existing literature by providing evidence on how modified‐atmosphere fermentation with CO_2_ influences both the chemical and sensory quality of natural coffees under real production conditions. Unlike previous research mainly focused on laboratory trials or wet fermentation systems, with this work it is possible to show how controlled CO_2_ has an influence on natural coffee quality. The findings will broaden the current understanding of fermentation biochemistry in dry‐processed coffees and offer practical guidance for producers seeking to standardize natural profiles and reduce quality variability in the field.

## 2. Materials and Methods

### 2.1. Plant Material

Red coffee cherries (*Coffea arabica* cv. ‘Colombia’) were harvested simultaneously at a ripening Stage 5–6 from a productive lot at the Santa Helena farm, following the criteria described by Peñuela et al. [12] (Figure 1a). The farm is situated at an altitude of 1740 meters above sea level on the eastern slope of the Western Cordillera in the municipality of El Águila, Valle del Cauca, Colombia.

Figure 1Initial processing of red coffee cherries cv. ‘Colombia’. Harvesting at a ripening Stage 5–6 (a) and after flotation (b).

**Figure:**
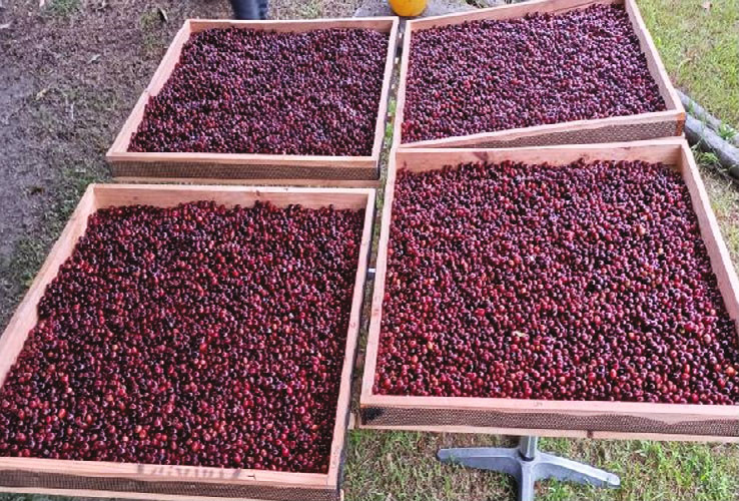
(a)

**Figure:**
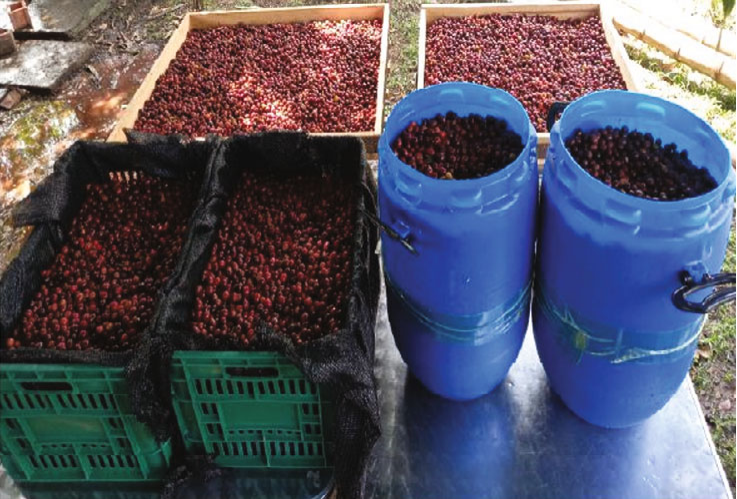
(b)

### 2.2. Coffee Cherry Processing and Fermentation Methods

After harvesting, the coffee cherries were transported to the Centro de Tecnologías Agroindustriales (CTA), SENA, in Cartago, Valle del Cauca. The fruits were placed in four 60 L containers, each holding approximately 35 kg (Figure 1b), and the transport lasted 1.5 h. The containers were covered with lids featuring small holes to allow air circulation. During transportation, the cherries were protected from direct sunlight and maintained at ambient temperature (18°C–23°C). The average harvesting time was 3.5 h, resulting in a total of approximately 5 h between the beginning of harvest and the initiation of fruit selection at the destination site. Cherries were selected and sorted by flotation in potable water storage tanks [6, 13]. Fruits that floated (density < 1.0 g/cm^3^) or showed visual defects (e.g., inadequate ripeness or physical damage) were discarded following the methodology of Peñuela et al. [12].

Each fermentation method involved a volume of 30 L of ripe coffee cherries and involved the following treatments: fermentation in baskets exposed to air for 24 (P1) and 48 h (P2), fermentation in sealed containers with CO_2_ for 24 (P3) and 60 h (P4), and a control group (P0), following the procedures of Sánchez‐Riaño et al. [14] with some modifications. All treatments were carried out at room temperature (22°C–29°C). For treatments P3 and P4, the air inside the containers was displaced by in situ–generated CO_2_, which was introduced from the base of the container, whereas the displaced air escaped through a one‐way valve at the top. A total of 20 L of CO_2_ was produced by reacting 80 g of sodium bicarbonate (NaHCO_3_) with 1000 mL of 5% (v/v) acetic acid (CH_3_COOH) in a gas‐generating system.

After completing the fermentation period for each method, the coffee cherries were placed on individual trays in a solar coffee dryer operating at 24°C–42°C. To ensure uniform drying, the cherries were stirred three times daily. A system incorporating a dehumidifier was used to reduce the relative humidity (RH) between 30% and 55%, allowing for continuous drying until the cherries reached a moisture content of 10%–12% on a wet basis (wb). Under these conditions, the total drying time was 213 h. The moisture content of the coffee cherries was monitored throughout the drying process using a KETT PM‐450 electrical resistance moisture analyzer (Kett Electric Laboratory, Japan). After drying, the samples were stored in plastic packaging for 30 days prior to conducting the chemical and sensory analyses.

### 2.3. Chemical Analysis of Natural Coffee

The chemical evaluations were conducted at the laboratories of Centro de la Innovación, la Agroindustria y la Aviación, Rionegro, Antioquia, Colombia.

#### 2.3.1. Total Sugar Content

The coffee samples were ground and sieved. Then, 1.5 g of each sample was placed in a 50 mL flask, and 30 mL of water was added. The mixture was subjected to an ultrasonic bath at 60°C for 15 min [15]. After cooling, 1 mL of the solution was taken and purified using a STRATA‐U‐C18 solid phase extraction cartridge (Phenomex, Torrance, California, United States). The purified extract was then transferred to a 10 mL flask and mixed with a mobile phase of 75:25 v/v acetonitrile and water.

Quantification was performed using a calibration curve standard ranging from 40 to 500 *μ*g/mL for sucrose, glucose, and fructose. The analysis was performed with a Thermo Ultimate 3000 UHPLC system (Waltham, Massachusetts, United States) equipped with a charged aerosol detector (CAD) set to 5 Hz and 35°C [16]. The column temperature was maintained at 30°C, and the mobile phase consisted of 77:23 v/v acetonitrile and water. The flow rate was set to 1 mL/min, with an injection volume of 20 *μ*L. The total reading time was 25 min. Results were expressed as g/100 g of sample.

#### 2.3.2. Extraction of Lipids and Determination of Fatty Acids Composition

The samples were ground, and moisture was removed by drying them in an oven at 103°C for 1 h. Using an automatic Soxhlet solid‐liquid extraction system, 5 g of each dried sample was subjected to extraction with hexane at a reflux temperature for 2 h. After the extraction, the hexane was evaporated, and the fat mass was calculated as the difference between the mass of the empty extraction vessel and its mass after the procedure.

The fatty acid profile was determined according to the NTC 4967 standard [17]. Fatty acids were analyzed using a gas chromatograph (7890A; Agilent Technologies, Santa Clara, California, United States) equipped with a flame ionization detector (FID) considering helium as the carrier gas (30 mL/min). The separation of the compounds was performed with a ZB‐FAME column (100 m × 0.25 mm × 0.20 *μ*m; Phenomenex). Each sample was injected at a volume of 1 *μ*L, the injector temperature was 250°C, and the initial purge flow rate was 3 mL/min. A temperature of 40°C was maintained for 2 min, then the temperature was increased at 60°C/min to 80°C and held for 1.5 min, followed by 40°C/min to 160°C, and 5°C/min to 185°C, and finally heated at 30°C/min–260°C and held for 2 min. The results were presented as the percentage of fatty acids composition (%).

#### 2.3.3. Chlorogenic Acid Content

The chlorogenic acid content was performed according to Velkoska‐Markovska et al. [18]. Three extractions were made. For the first and second extraction, 1.5 g of each sample were added to 25 mL of water, then heated and boiled for 5 min with continuous mixing. For the third extraction, the proportion of sample and solvent was maintained, but the solvent was changed for methanol. Subsequently, the mix was ultrasonicated for 15 min. Separation was achieved on a Poroshell 120 EC‐C18 (50 mm × 3 mm; 2.7 *μ*m) column (Agilent) with a mobile phase consisting of water with 1% H_3_PO_4_ and acetonitrile (90:10 v/v). The analysis was performed using isocratic elution with a flow rate of 1 mL/min, and a volume of 5 *μ*L was injected. A wavelength of 325 nm was set up for UV detection. The retention time of chlorogenic acid was 0.96 min, whereas the time of the analyses was less than 1 min.

### 2.4. Sensory Analysis of Coffee

The coffee samples were analyzed in the laboratories of Escuela Nacional de la Calidad del Café (ENCC; Armenia, Quindío, Colombia). For this purpose, dried coffee cherries (10%–12% moisture content) were processed using a roller mill adjusted to 2.0–2.5 mm to obtain green coffee. Defective beans were manually sorted out, and 110 g of samples were roasted 24 h using a probat sample roaster. Sensory quality attributes of the coffee were evaluated according to the protocol of the Specialty Coffee Association (SCA), following the 100‐point scale system. For cupping, 8.25 g (±0.25 g) of medium‐coarse ground coffee per cup, brewed with 150 mL of water at 93°C (coffee‐to‐water ratio of 1:18.18). Each sample was prepared in five replicate cups. The sensory evaluation was performed by a panel of three certified Q‐graders assessing fragrance/aroma, flavor, aftertaste, acidity, body, balance, uniformity, clean cup, sweetness, and overall quality.

### 2.5. Statistical Analysis

A completely randomized design was employed. After the coffee drying process was completed, five samples (600 g per treatment) were randomly collected from each treatment for sensory analysis, which was performed in triplicate (5 × 5 × 3), resulting in a total of 75 sensory evaluations. For the chemical analyses, three samples (30 g each) were randomly taken from each process, and analyses were also performed in triplicate (5 × 3 × 3), for a total of 45 chemical determinations. Principal component analysis (PCA), correlation, and correspondence analysis were performed to analyze the variables. Additionally, an analysis of variance (ANOVA) was conducted for the sensory evaluation variables (*p* < 0.05). All the analyses were performed in *R* (Indianapolis, Indiana, United States) with the FactoMineR package.

## 3. Results and Discussion

### 3.1. Total Sugar and Chlorogenic Acid Content of Natural Coffee

**Figure 2:**
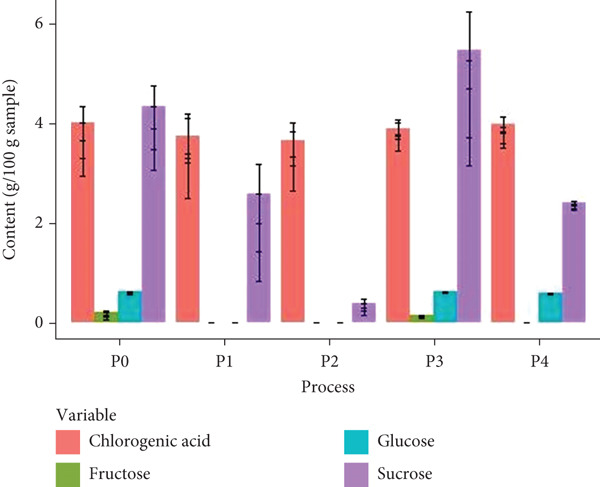
Total sugar and chlorogenic acid content of dry‐processed coffee using five different fermentation methods (*P*0 = control, *P*1 = aerobic fermentation for 24 h, *P*2 = aerobic fermentation for 48 h, *P*3 = fermentation with modified atmosphere for 24 h, *P*4 = fermentation with modified atmosphere for 60 h). The vertical lines represent standard deviation.

The analysis primarily focused on reducing sugars (fructose, glucose, and sucrose) in the samples from the five treatments. According to Figure 2, all samples exhibited a high content of sucrose, followed by glucose. However, sucrose consumption during fermentation was particularly notable in P2 and P4, likely due to its utilization as a substrate during the extended fermentation periods (Figure 2). Conversely, in P3, the utilization of sugars, mainly sucrose, by the microorganisms involved in fermentation was slower and somewhat inhibited by the presence of CO_2_. According to da Silva Vale et al. [19], the presence of residual sugars during spontaneous fermentations is possible, as has been reported in our country. Additionally, the indigenous microbiota can metabolize the sugars present in the mucilage, particularly fructose, through the activity of microorganisms with a fructophilic phenotype.

Another study showed that coffee processing affects sugar content. Knopp et al. [20] exposed coffee cv. Acaiá to wet processing (mechanical pulping, underwater fermentation in a tank for 22 h, solar drying for 6 days), dry processing (drying coffee cherries for 16 days), and semidry processing (mechanical pulping and drying of wet and mucilaginous parchment beans). The sucrose content in samples from different processing methods averaged 8 g/100 g, whereas glucose and fructose contents were less than 0.05/100 g. Compared with our study, the sucrose content was higher because no fermentation was carried out, although the glucose and fructose contents were similar.

Chlorogenic acid is one of the primary phenolic compounds in coffee [21]. As shown in Figure 2, fermentation did not significantly affect the chlorogenic acid content in natural coffees, although the lowest levels were observed in the samples from the P1 and P2 treatments. The exposure to air and environmental conditions in these treatments may have contributed to the reduction of this bioactive compound due to thermal degradation and isomerization [22]. In contrast, the P3 and P4 treatments seem to promote the production of bioactive compounds that offer significant health benefits. Generally, fermentation is associated with the release of free and conjugated phenolics through biotransformation, improving their digestibility and bioactivity in the human body [23, 24]. Furthermore, the content of organic acids, including chlorogenic acid, is influenced by the composition of the microbial community present during fermentation. The presence of yeasts such as *Saccharomyces cerevisiae* has been shown to increase the levels of this compound [25]. Similarly, Pereira Bressani et al. [26] reported an increase in chlorogenic acid content in coffee cherries fermented for 72 h in closed polyethylene containers inoculated with different yeast species. Therefore, fermented natural coffee may offer a better cost–benefit ratio compared with other processing methods.

### 3.2. Fatty Acids Composition of Natural Coffee

**Figure 3:**
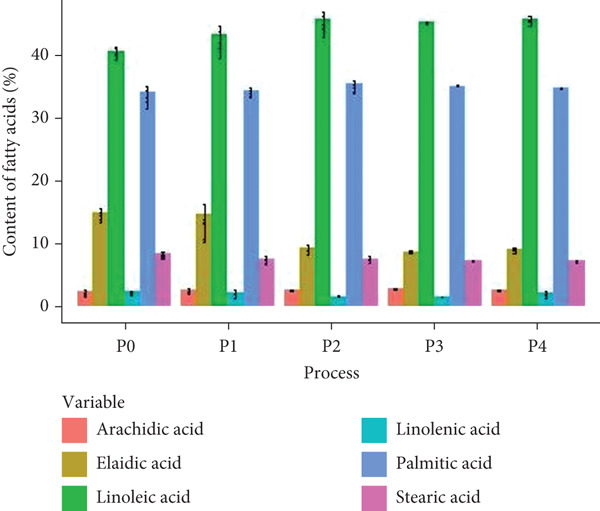
Fatty acids composition (%) of dry‐processed coffee using five different fermentation methods (*P*0 = control, *P*1 = aerobic fermentation for 24 h, *P*2 = aerobic fermentation for 48 h, *P*3 = fermentation with modified atmosphere for 24 h, *P*4 = fermentation with modified atmosphere for 60 h). The vertical lines represent standard deviation.

The analysis primarily focused on arachidic, elaidic, linoleic, linolenic, palmitic, and stearic acids (Figure 3). No significant differences were observed in the content of arachidic, linolenic, palmitic, and stearic acids across the treatments (*p* > 0.05). The P2, P3, and P4 treatments showed the highest linoleic acid content. While fatty acid composition is influenced by various factors, including cultivar, geographical origin, and processing methods, linoleic acid is typically the predominant unsaturated fatty acid in coffee, comprising approximately 40%–45% of the total fatty acids [27]. In contrast, the linoleic acid content in the P0 and P1 treatments was lower, likely due to metabolic processes, microbiological and enzymatic activity, and lipoxidation during fermentation and drying [28].

For elaidic acid, treatments P0 and P1 exhibited the highest content values (> 10%). This compound is a trans isomer of oleic acid that can negatively affect coffee quality when present in high concentrations [29]. Alabdalall [30] noted that increased levels of elaidic acid in coffee can reduce acidity, a key sensory attribute. In our study, the samples with the highest elaidic acid content also recorded the lowest final scores and acidity in the sensory analysis. Similarly, Pereira Figueiredo et al. [31] observed a negative correlation between elaidic acid and sensory quality in four genotypes of *Coffea arabica* L. subjected to semidry processing, with higher‐quality coffee beans showing lower elaidic acid content.

### 3.3. Sensory Attributes of Natural Coffee

The sensory evaluation revealed no statistical differences for uniformity, clean cup, and sweetness, as all treatments received a score of 10 points, the highest rating on the SCA affective scale (*p* > 0.05). This confirms that, under the conditions of this study, none of the fermentation methods generated cup defects across the five treatments and their respective repetitions. Conversely, ANOVA demonstrated significant statistical differences for fragrance/aroma (*F*/*A*), flavor, remaining flavor, acidity, body, balance, taster score (T.S.), and final score (*p* < 0.05). For the variable *F*/*A*, treatments P2, P3, and P4 exhibited the best performance and were statistically different from P0 and P1, which showed the lowest average values (Table 1).

**Table 1:** Mean values for sensory quality attributes across the five fermentation methods.

**Variable**	**P1**	**P2**	**P3**	**P4**	**P0**
*F*/*A*	7.70^bc^	8.00^ab^	8.15^ab^	8.30^a^	7.45^c^
Flavor	7.90^ab^	7.90^ab^	8.05^a^	8.15^a^	7.50^b^
Remaining flavor	7.75^ab^	7.90^a^	7.95^a^	7.75^ab^	7.35^b^
Acidity	7.90^ab^	7.85^ab^	8.20^a^	8.00^a^	7.50^b^
Body	7.85^bc^	7.75^c^	8.05^ab^	8.15^a^	7.50^d^
Balance	7.75^b^	7.75^b^	8.20^a^	8.15^a^	7.45^b^
T.S.	7.85^ab^	7.95^a^	8.30^a^	8.00^a^	7.40^b^
Final score	84.70^c^	85.10^bc^	86.90^a^	86.50^ab^	82.15^d^

*Note:* Means with a letter in common are not significantly different (*p* > 0.05).

Table 1 also revealed that flavor and acidity displayed similar behavior, with significant statistical differences between P0 and P3–P4 (*p* < 0.05), where the latter showed better performance. For remaining flavor, significant differences were observed only between P0 and P3, with P3 presenting the best result. The variable body exhibited statistical differences between P0 and the other treatments, whereas P4 was statistically different from P1 and P2, as well as differences observed between P2 and P3. In the case of balance, two distinct groups emerged: one comprising P0, P1, and P2, and the other consisting of P3 and P4, which showed higher values. For the T.S., statistical analysis indicated no significant differences between P1, P2, P3, and P4, nor between P1 and P0 (*p* > 0.05). The variable final score, which represents the sum of all sensory attributes, showed that P0 was statistically different from the other treatments. Furthermore, P1 and P2 were statistically different from P3 and P4, with the latter treatments achieving the highest scores in the sensory evaluation based on the SCA scale.

On the other hand, the values of final score for P3 and P4 overcame those reported in literature. Ferreira et al. [32] analyzed coffee samples from the southwestern Bahia municipalities of Barra do Choça and Encruzilhada in Brazil, harvested during the 2007/2008 season, which were subjected to natural processing. The final scores of these samples averaged 78.12 and 75.65, respectively. Therefore, the results of our study could be attributed to the fact that lactic and acetic acid bacteria are highly susceptible to CO_2_, which enables better control of acidity levels and may reduce certain remaining flavors [7].

### 3.4. PCA, Correlation, and Correspondence Analysis of Chemical Parameters and Sensory Attributes of Natural Coffee

**Figure 4:**
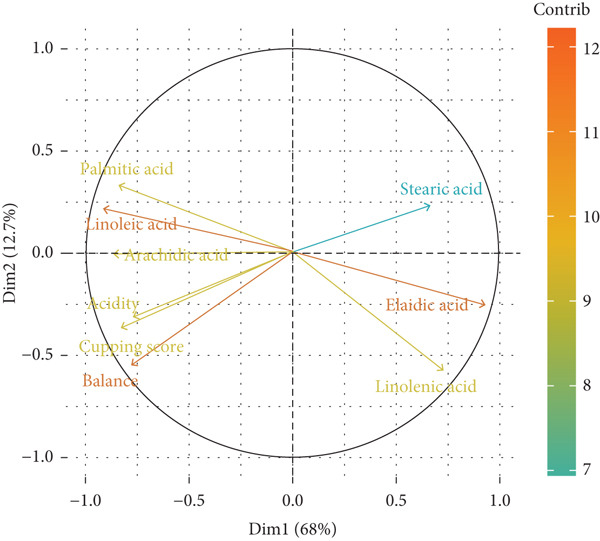
Factor analysis of the principal fatty acids and sensory attributes of natural coffee subjected to five different fermentation treatments.

PCA and correlations are observed between sensory attributes (acidity, balance, T.S., and final score) and fatty acids composition variables (palmitic, stearic, elaidic, linoleic, linolenic, and arachidic acids) (Figure 4). The first principal component (Dim1), which explains 68% of the variance, divides the variables into two distinct groups. On the right side, stearic, elaidic, and arachidic acids are positively correlated with each other but negatively correlated with the sensory quality attributes (acidity, balance, T.S., and final score) located on the left side. This suggests that higher concentrations of these fatty acids are linked to lower sensory quality. Conversely, on the left side, linoleic and linolenic acids are positively correlated with sensory quality attributes, indicating that increased levels of these fatty acids contribute to improved sensory properties in coffee. Therefore, coffee processing control incises significantly on sensory attributes [33].

The second principal component (Dim2), which accounts for 12% of the variance, provides additional insights into the relationships among the variables. Palmitic acid is positioned distinctly, suggesting it exhibits a unique relationship with sensory attributes compared with the other fatty acids. The clustering of sensory quality attributes (acidity, balance, T.S., and final) highlights their strong interrelation and positive correlation, indicating that an improvement in one sensory attribute is likely to enhance the others. These findings emphasize the significant role of the fatty acid profile, particularly the levels of linoleic acid and linolenic acid, in influencing the sensory quality of coffee. In contrast, higher concentrations of stearic acid, elaidic acid, and arachidic acid are associated with diminished sensory quality [28].

**Figure 5:**
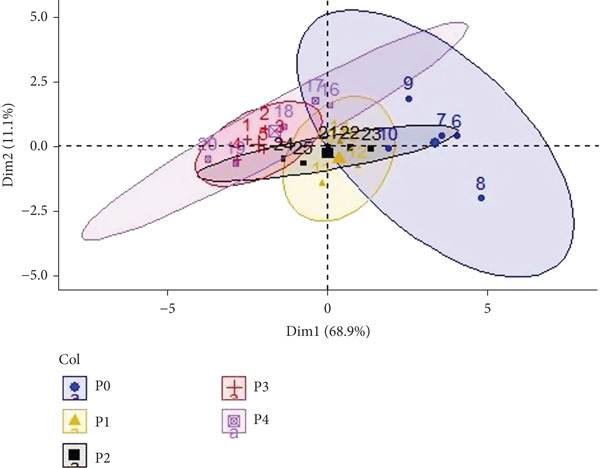
Factor analysis of the sensory attributes of natural coffee subjected to five different fermentation treatments.

Another PCA was conducted using sensory analysis variables, including fragrance/aroma, flavor, flavor/aftertaste, acidity, body, balance, and final score for the various processing methods (*P*). The resulting biplot illustrates the correlations among sensory attributes and the grouping of the different processing methods (Figure 5). The first principal component (Dim1), accounting for 68.9% of the variance, distinctly separates the treatments along the horizontal axis. P0, P1, and P2 treatments are grouped on the left, whereas processes P3 and P4 are positioned on the right, highlighting significant differences in sensory characteristics between these two groups. The second principal component (Dim2), contributing an additional 11% of the variance, provides further distinction among treatments. Along this vertical axis, a clearer separation of P0, P1, and P2 is evident. This analysis effectively differentiates the sensory profiles associated with the various coffee processing methods, demonstrating their distinct impacts on sensory attributes.

**Figure 6:**
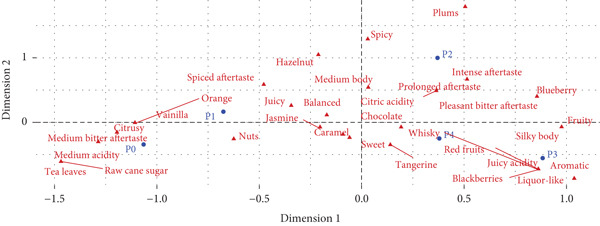
Correspondence analysis of the sensory attributes of natural coffee subjected to five different fermentation treatments.

The correspondence analysis depicted in Figure 6 illustrates how the main sensory descriptors of coffee are grouped and interrelated. Along the horizontal axis (Dimension 1), the attributes representing flavors, aromas, and characteristics of coffee are differentiated. On the far right, descriptors such as plums, spicy, intense aftertaste, and blueberry are located, whereas on the far left, descriptors like tea leaves, raw cane sugar, and medium acidity are positioned. Meanwhile, vertical (Dimension 2) further separates the descriptors vertically. In the upper section, attributes such as silky body, medium body, and balanced are clustered, whereas the lower section includes descriptors like whisky, bitter, and liquor‐like.

Based on the positioning of the different processes, distinct sensory profiles were observed. P0 samples were positioned near descriptors such as tea leaves, raw cane sugar, and medium acidity, suggesting that this process produces coffees with softer, more balanced, and delicate sensory profiles. P1 treatment generated some attributes like hazelnut, juicy, and balanced, indicating that this method generates coffees with intermediate characteristics, without any strong dominance of intense notes. P2 samples were positioned near descriptors such as intense aftertaste, blueberry, and pleasant bitter aftertaste, suggesting that this process is associated with the development of sweeter, spicier, and citrus flavors. P3 was associated with descriptors such as aromatic, liquor‐like, and silky body, indicating that this process results in coffees with more complex profiles, featuring intense flavors and fruity notes. P4 samples were located near descriptors like silky body, whisky, and liquor‐like, suggesting that this treatment produces coffees with a silkier structure, but also with more bitter characteristics. These positions indicate how each processing method influences the sensory profiles of the resulting natural coffees.

## 4. Conclusions

This study found that fermentation processes involving CO_2_ demonstrated better sensory performance compared with treatments fermented in contact with ambient air for natural coffee production. The fermentation and drying processes implemented in this research resulted in natural coffees free of sensory defects. The highest concentrations of linoleate were associated with higher sensory scores, whereas elevated levels of elaidic acid correlated with lower sensory ratings. For future investigations, it is essential to conduct chemical analysis evaluations that encompass a broader range of compounds to better explain the differences observed in the sensory analysis.

## Conflicts of Interest

The authors declare no conflicts of interest.

## Funding

No funding was received for this manuscript.

## Data Availability

The data that support the findings of this study are available from the corresponding author upon reasonable request.
